# Ventilatory Response to Exercise in HFrEF-COPD: Importance of Exercise Modality

**DOI:** 10.3390/jcm14082538

**Published:** 2025-04-08

**Authors:** Marta Íscar Urrutia, Julia Herrero Huertas, Marina Acebo Castro, Ramón Fernández Álvarez, Beatriz Díaz Molina, Marta García Clemente

**Affiliations:** 1 Department of Pneumology, Asturias Central University Hospital, 33011 Oviedo, Spain; martaiscar@gmail.com (M.Í.U.); marinaacebo@hotmail.es (M.A.C.); enelllano@gmail.com (R.F.Á.); mgclemen@gmail.com (M.G.C.); 2 Department of Pneumology, Fundación Jiménez Díaz University Hospital, 28040 Madrid, Spain; 3 Department of Cardiology, Asturias Central University Hospital, 33011 Oviedo, Spain; beadimo@gmail.com

**Keywords:** heart failure, COPD, cardiopulmonary exercise test, cycle ergometer, treadmill

## Abstract

**Background:** Heart failure with reduced ejection fraction (HFrEF) frequently coexists with chronic obstructive pulmonary disease (COPD), and both conditions share symptoms such as exertional dyspnea. The cardiopulmonary exercise test (CPET) is an essential tool for assessing ventilatory and cardiovascular function and plays a key role in the differential diagnosis of dyspnea. However, the impact of exercise modality on the ventilatory and cardiovascular parameters obtained remains unclear in these groups. Our aim is to compare the oxygen consumption (V^·^O_2_) and breathing reserve (BR) values obtained from CPET on a treadmill and a cycle ergometer in patients with HFrEF-COPD and those with HFrEF alone. **Methods:** A prospective observational study included 65 patients with HFrEF (LVEF ≤ 40%), 18 of whom had COPD. Two CPETs were performed, the first on a treadmill and the second 48–72 h later on a cycle ergometer. **Results:** In the group with HFrEF-COPD, peak oxygen consumption (VO_2_/kg) and maximum ventilation (VE) values were significantly higher on the treadmill (20 ± 5 vs. 17 ± 4 mL/kg/min, *p* < 0.001 and 55 ± 19 vs. 45 ± 11 L/min, *p* < 0.001, respectively), while breathing reserve (BR%) was lower on the treadmill (16 ± 21 vs. 33 ± 20, *p* < 0.001). Compared to the HFrEF group, patients with HFrEF-COPD had a lower BR in both exercise modalities (*p* = 0.01). **Conclusions:** Treadmill CPET demonstrates greater oxygen consumption and a more pronounced ventilatory response. BR is consolidated as a differential parameter in ventilatory limitation. The choice of exercise modality should be considered based on the underlying pathologies and the objective of the test.

## 1. Introduction

Heart failure with reduced ejection fraction (HFrEF) is a highly prevalent condition with significant morbidity and mortality, characterized by the marked impairment of cardiac function and patient quality of life. Chronic obstructive pulmonary disease (COPD) frequently coexists with HFrEF, largely due to shared risk factors such as smoking, advanced age, and a sedentary lifestyle. Oxidative stress, chronic systemic inflammation, and hyperactivity of the sympathetic nervous system are pathogenic mechanisms shared by both conditions [1].

The coexistence of COPD worsens the prognosis of HFrEF, increases clinical complications, and presents additional challenges in diagnosis. According to studies carried out with different European cohorts, the prevalence of COPD varies from 9 to 41% among patients with heart failure. Conversely, the prevalence of heart failure increases significantly in hospitalized COPD patients, reaching up to 70% in those requiring mechanical ventilation [2].

Dyspnea on exertion is a predominant symptom shared by both conditions, posing a diagnostic challenge in distinguishing the underlying cause in patients with concomitant COPD and HFrEF [3]. Cardiopulmonary exercise testing (CPET) is valuable for functional assessment, prognosis, and exercise prescription across various pathologies. It is especially useful for the differential diagnosis of dyspnea, given its capacity to evaluate both the cardiocirculatory and ventilatory systems [4].

Exercise intolerance in HFrEF is based on an alteration of both the supply of oxygen to skeletal muscle and the alteration of its utilization by myocytes. This imbalance between oxygen supply and demand becomes evident during progressive exercise testing, leading to a reduced maximal workload. This limitation reflects the lack of enough adenosine triphosphate to maintain skeletal muscle contraction, with a primary manifestation being reduced oxygen consumption (V^·^O_2_) [5]. V^·^O_2_ is widely recognized as the gold standard for evaluating functional capacity.

In COPD, reduced ventilatory capacity is characterized by diminished breathing reserve (BR) at maximum exercise, which results in a decrease in functional capacity, manifested by a decreased peak V^·^O_2_. In contrast, patients with HFrEF typically preserve ventilatory reserve, making BR a clinically significant variable for distinguishing between cardiac and pulmonary disorders [5].

To improve exercise intolerance, in addition to pharmacological treatment and devices (pacemakers, endobronchial valves…), exercise training for both pathologies must be taken into account [6,7]. The most common exercise modalities are the cycle ergometer and the treadmill with a wide variety of protocols [5]. Both exercise modalities present distinct advantages. The treadmill induces greater systemic stress on the cardiovascular and ventilatory systems, while the cycle ergometer allows for the precise quantification of external work [8,9,10]. Our group carried out a comparative study of both ergometers in 65 patients with HFrEF, demonstrating a 20% higher V^·^O_2_ max with better ventilatory efficiency and lower BR for the treadmill compared to the cycle ergometer, using protocols of a similar exercise load and test duration [11].

The limited studies conducted on patients with HFrEF-COPD have been exclusively performed using a cycle ergometer. These studies showed that patients with both conditions, despite having similar resting limitations, exhibit lower exercise tolerance compared to those with either COPD or HFrEF alone [12,13,14,15], without considering the differential impact of the exercise modality on assessing functional limitation. Our research fills this gap by directly comparing ventilatory and cardiovascular responses during treadmill and cycle ergometer cardiopulmonary exercise testing.

We hypothesize that the choice of exercise modality, whether treadmill or cycle ergometer, plays a critical role in assessing whether exercise limitation is predominantly cardiac or ventilatory in patients with HFrEF-COPD, as it significantly influences the ventilatory and cardiovascular parameters obtained. The primary aim of this study was to evaluate the variables obtained from CPET performed on a treadmill and a cycle ergometer in patients with HFrEF alone and HFrEF-COPD.

## 2. Materials and Methods

A prospective study was conducted on patients diagnosed with chronic HFrEF referred for functional assessment in the Exercise Unit from the Advanced Heart Failure and Heart Transplant Unit of the Central University Hospital of Asturias between July 2019 and March 2020. Medical follow-up was conducted until October 2024.

All patients had a documented diagnosis of chronic heart failure with reduced ejection fraction (≤40%) using the criteria of the ESC 2021 [6] in a stable situation. Patients with spirometry with a FEV1/FVC ratio < post-bronchodilator LIN [16] were classified as obstructive, and the diagnosis of COPD was considered when the criteria of the GOLD 2019 guideline were met [17].

Exclusion criteria included an inability to perform stress tests, hospitalization, chronic respiratory failure, disease exacerbation, or uncontrolled atrial fibrillation (AF) within three months before study enrollment.

The study was approved by the Research Ethics Committee of the Principality of Asturias with registration number N174/19. All patients included in the study received the study information sheet and signed the consent form accepting their participation in the study.

Each patient underwent two CPETs performed to their maximum tolerance. The first CPET was conducted on a treadmill (HP Cosmos Pulsar 2002, Nußdorf, Germany) using a modified version of the Bruce protocol [18]. This protocol consisted of 1 min stages with progressively increasing speeds ranging from 2.7 to 6 km/h and an incline gradient rising from 0% to 16%. The second CPET was carried out 72 h later using a cycle ergometer (Corival Lode BV, Groninga, The Netherlands), with workload increments of 5–20 W/min adjusted to the functional limitation of each patient. Before the second test, a clinical evaluation confirmed the absence of changes in symptoms, physical findings, body weight, or medication. Test termination criteria included the maximum perceived effort reported by the supervised patient, the occurrence of severe cardiovascular events, or the presence of limiting symptoms. Monitoring was extended into the recovery phase, which lasted 3 min [19].

Demographic, anthropometric, and clinical data, such as smoking status, comorbidities, treatment, NYHA functional classification, dyspnea (mMRC), and echocardiography findings, were collected. Standard respiratory and cardiorespiratory parameters were measured on a breath-by-breath basis during the two CPETs, following ATS/ACCP standards [19,20].

**Figure 1:**
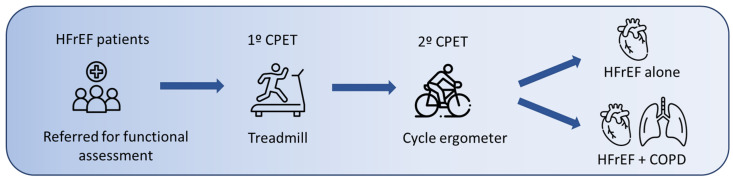
Study design. Included patients underwent two cardiopulmonary exercise tests (CPET): the first on a treadmill and the second on a cycle ergometer. Participants were categorized into two groups based on comorbidities: HFrEF alone and HFrEF-COPD.

Data from the HFrEF-COPD group were analyzed and subsequently compared with the HFrEF-alone group (Figure 1). This subanalysis has not been previously published.

### Statistical Analysis

Statistical analysis was performed using Stata v15.4.2 (Stata Corp LLC., College Station, TX, USA). Quantitative variables were expressed as the mean ± standard deviation (SD) for those following a normal distribution and as the median ± interquartile range (IQR) for those not following a normal distribution, while qualitative variables were presented as percentages. Initially, a descriptive analysis of the sample was carried out. The normality of the sample distribution was assessed using the Shapiro-Wilk test. For variables following a normal distribution, the Student’s *t*-test was applied for mean comparison, whereas the Wilcoxon test and the U–Mann–Whitney test was used for variables that did not meet the normality assumption. Categorical variables were analyzed using the chi-square test or Fisher’s exact test, when applicable. To assess the correlation of variables, the Pearson correlation coefficient was used. A value of *p* < 0.05 was considered statistically significant.

## 3. Results

### 3.1. Sample Characteristics

Sixty-five patients with HFrEF were included, 18 of whom had coexisting COPD. Table 1 presents the general characteristics of both groups (patients with and without COPD).

The HFrEF-COPD subgroup had a higher mean age than the group without COPD (61.6 ± 7.6 vs. 56.4 ± 9.6 years; *p* = 0.04) and a lower BMI (27.1 ± 4.4 vs. 30.0 ± 4.2 kg/m^2^; *p* = 0.01). There were no significant differences in LVEF or sex distribution (Table 1).

According to the GOLD classification, 8 patients (44.4%) in the HFrEF-COPD group had mild COPD, 9 (50%) had moderate COPD, and 1 patient (5.5%) had severe COPD. 94% of patients in this group had a history of smoking compared to 68% of patients without COPD. As expected, lung function was significantly lower in the HFrEF-COPD group.

Regarding the functional class, in the HFrEF-COPD group, we observed a slight increase in patients in the NYHA class II compared to the group without COPD. Regarding the etiology of heart failure, ischemic heart disease was more prevalent in the HFrEF-COPD group (56% vs. 39%), followed by idiopathic etiology (39% vs. 44%).

The treatment of heart failure in both groups is detailed in Table 2.

Patients in both groups were treated with beta-blockers and Renin-Angiotensin-Aldosterone System inhibitors (RAASi) with different diuretic combinations. There were no significant differences in the treatment of both groups except with implantable cardioverter defibrillators.

Regarding inhaled treatment, only four patients (22.2%) used inhaled anticholinergics and two (11.1%) used inhaled corticosteroids. The remaining patients (88.8%) used inhaled treatments as needed, such as salbutamol, LABA + LAMA, or LAMA.

The percentage of patients with an implantable cardioverter-defibrillator (ICD) was significantly higher in the HFrEF-COPD group compared to the percentage of patients without COPD (77.8% vs. 40.4%).

### 3.2. Cardiopulmonary Exercise Test (CPET), Group HFrEF-COPD

The time interval between the two CPETs performed was 3.0 ± 1.9 days. The parameters are detailed in Table 3.

#### 3.2.1. Effort Level

The exercise duration and maximum respiratory ratio were similar in both exercise modalities. The RQ achieved in both protocols (0.9 (IQR: 0.9–1) vs. 1 (IQR: 0.9–1) (*p* = 0.880)) did not show significant differences, suggesting a similar degree of effort.

The early interruption of the test was necessary in two patients (11.1%) during the treadmill test due to dyspnea, while in the cycle ergometer, it was suspended in five patients (27.7%) due to fatigue in the lower limbs. In total, seven patients (38.9%) finished the test before reaching the ventilatory threshold (VT).

#### 3.2.2. Oxygen Consumption (V^·^O_2_)

In the behavior of V^·^O_2_ throughout the CPET, there were no differences between both modalities in basal consumption (subject at rest); however, a significant difference was evident both in VT and in the maximum exercise point reached (V^·^O_2_ peak) (*p* < 0.001).

The peak V^·^O_2_kg value (mL/kg/min^−1^) was significantly higher in the treadmill test compared to the cycle ergometer: 20 (IQR: 17–23) vs. 17 (IQR: 15–19); *p* < 0.001).

A difference of 18.9% in the V^·^O_2_ peak and 18% in the V^·^O_2_/kg peak was observed in favor of the treadmill (*p* < 0.001). Both parameters showed an excellent correlation between the two tests (r = 0.93 y r = 0.89, respectively; *p* < 0.001).

#### 3.2.3. Ventilatory Response and Gas Exchange

A more pronounced ventilatory response was observed on the treadmill, with a higher maximum respiratory rate ((RR) 30.9 (IQR: 26.5–36.5) vs. 25.5 (IQR: 21–28.5) bpm; *p*_0.017)) and maximum ventilation ((V^·^E) 59 (IQR: 49–62.5) vs. 45.9 (IQR: 38.6–46.8) L/min; *p* < 0.001)). Although EqO_2_ and EqCO_2_ were higher on the treadmill (38 (IQR: 34–39) vs. 36.5 (IQR: 33–39) and 38 (IQR: 35.5–39) vs. 37 (IQR: 33.5–39.5), respectively), these differences did not reach statistical significance.

The baseline P_ET_CO_2_ difference minus the threshold P_ET_CO_2_ was higher in the cycle ergometer without reaching statistical significance.

The lower V^·^E Slope (32 (IQR: 29–37) vs. 35 (IQR: 31–38.6); *p* = 0.042) on the treadmill reflects better ventilatory efficiency in this exercise modality.

It should be noted that the BR at the maximum effort achieved was significantly lower on the treadmill (12 (IQR: 2–35) vs. 25.5 (IQR: 22–48) 3; *p* < 0.001)), which demonstrates a greater degree of air trapping in standing.

Oscillating ventilation (EOV) was not observed in any patient on the treadmill and was only identified in two patients (11.1%) on the cycle ergometer.

No desaturation was observed during exercise in any of the exercise modalities.

#### 3.2.4. Cardiovascular Response

The maximum HR was higher on the treadmill (119 (IQR: 102.5–132) vs. 108.5 (IQR: 100–120.5)) without reaching statistical significance. The heart rate recovery rate was similar for both ergometers in the 14 patients in sinus rhythm.

Regarding the blood pressure response, starting from similar baselines, the systolic blood pressure response was greater on the treadmill (170 (IQR: 150–185) vs. 162.5 (IQR: 140–180)) without reaching statistical significance, unlike thaat of diastolic blood pressure, which reached higher values on the cycle ergometer (82.5 (IQR: 80–90) vs. 80 (IQR: 70–90) (*p* = 0.037)), reaching statistical significance in this case.

The Oxygen Pulse (V^·^O_2_/FC) in maximum exercise reached higher values on the treadmill (14.1 (IQR: 11.9–17.7) vs. 12.7 (IQR: 10.3–15.5); *p* < 0.001)), which, together with the higher HR achieved, clearly indirectly reflects a greater cardiac output in the exercise performed on the treadmill.

### 3.3. Comparison of Patients with HFrEF with and Without COPD

When analyzing the results of both CPETs in both groups, we observed the absence of significant differences in most of the parameters studied (Table 4).

The maximum oxygen consumption (V^·^O_2_ peak) and V^·^O_2_/kg peak were slightly lower in patients with COPD, although the differences did not reach statistical significance. This could reflect a more pronounced functional limitation in this subgroup.

Patients with HFrEF-COPD presented significantly lower BR during maximum effort on both types of ergometers, although this was more pronounced on the treadmill (12% (IQR: 2–35) vs. 30% (IQR: 25.3–34.9); *p* = 0.006)) compared to the cycle ergometer (30% (IQR: 22–48) vs. 27% (IQR: 24.9–32.5); *p* = 0.02)). These results demonstrate the value of BR as a key differentiating characteristic between the two groups and reflect the greater ventilatory effort on the treadmill, associated with a higher air trapping degree when standing.

Regarding ventilatory efficiency, the HFrEF-COPD group presented worse efficiency in both types of exercise, reflected in a higher V^·^E slope, although it only acquired statistical significance in the cycle ergometer (35% (IQR: 31–38.6) vs. 32% (IQR: 27–35.8); *p* = 0.042)). Concerning oscillating breathing, this was observed more frequently in patients without COPD, both on the treadmill (41% vs. 0%; *p* = 0.001) and on the cycle ergometer (46% vs. 12%; *p* = 0.01).

There were no significant differences in maximum heart rate (HR), HR recovery index, or oxygen pulse, which reinforces the performance of a comparable effort level between both groups.

#### 3.3.1. Symptoms and Preference

Patients in both groups presented a greater degree of dyspnea on the treadmill and more pronounced lower limb fatigue on the cycle ergometer, with no significant differences between both groups.

Regarding the preference for the type of ergometer, the majority of patients in both groups opted for the cycle ergometer, due to the increased feeling of safety during its use.

#### 3.3.2. Mortality

During follow-up until October 2024, mortality was higher in the HFrEF-COPD group (27.8% vs. 6.4%).

Causes of mortality in patients with COPD included COVID-19 pneumonia (two cases) and cardiovascular causes (three cases). In the group of patients without COPD, three patients died due to lung neoplasia, COVID-19 pneumonia, and post-heart transplant complications.

## 4. Discussion

This is the first study that evaluates the physiological variables of exercise in patients with COPD coexisting with heart failure with reduced ejection fraction (HFrEF) using two types of ergometers, treadmill and cycle ergometer.

The main finding of this study is that the functional assessment of patients with HFrEF-COPD on a treadmill reveals increased oxygen consumption and an improvement in gas exchange, despite inducing greater air trapping in patients with airway obstruction.

Peak V^·^O_2_ is the most important variable of CPET, and it is usually decreased in both cardiac and respiratory pathology. Depending on the type of ergometer used, the difference in peak V^·^O_2_ is generally considered to be 5–10% greater on a treadmill [3]. In our study, with a similar effort level, there was a difference of 19.8% in peak V^·^O_2_/kg for HFrEF and of 18% in peak V^·^O_2_/kg for HFrFE-COPD, confirming the superiority of the treadmill, following previous literature, in healthy subjects and patients with or without COPD. Furthermore, in patients with mixed pathology, this higher V^·^O_2_ is maintained despite the coexistence of COPD. We did not find in the literature reviewed to date any studies that used both ergometers in mixed pathology (HFrEF-COPD).

The impact of the difference in the V^·^O_2_ obtained is determined by the prognostic scales in both groups. Prognostic assessment is of crucial importance in providing measures that improve quality of life and survival and reduce hospitalizations and healthcare costs [21].

Breathing reserve (BR) has been studied as a key parameter for distinguishing exercise limitation in patients with HFrEF-COPD. Our study confirms its usefulness, showing a significant decrease in BR in patients with COPD during exercise, secondary to hyperinflation and a lower maximum voluntary ventilation compared to patients without COPD [5].

In the functional assessment of patients with COPD, CPET on a treadmill is clearly recommended due to its superior sensitivity in detecting exertional desaturation and a greater decrease in BR [5]. Our findings reinforce this recommendation, demonstrating that the treadmill provides a more accurate characterization of ventilatory limitation than the cycle ergometer in these patients, making it a more effective tool for functional evaluation.

In our study, all patients with COPD had mixed disease with different degrees of ventilatory impairment (GOLD I–III). Despite the demonstrated benefits of bronchodilator treatment in patients with COPD, regardless of its association with heart failure [7], the poor adherence to medication prescribed for COPD compared to medication for HFrEF is striking. This finding, previously described in the literature [22], highlights the need for greater effort among pulmonologists and cardiologists in motivating treatment adherence.

The ventilatory response was greater on the treadmill, consistent with the study by Hsia et al. [23], which compared two linear protocols on a treadmill and cycle ergometer in 16 patients with COPD, noting lower BR and more pronounced desaturation on the treadmill. In our study, desaturation was not observed in any patient, probably due to the lower severity of the obstruction in our sample, with the behavior of V^·^E max being similar for both groups.

Ventilatory efficiency, assessed by V^·^E Slope, is a parameter widely used in clinical practice [5,21]. Both heart and lung diseases affect gas exchange at the lung level, which can be evaluated through the relationship or V^·^E to V^·^CO_2_ using a linear regression line. Our study demonstrated better ventilatory efficiency on the treadmill compared to the cycle ergometer, worsening for patients with mixed disease due to a greater deterioration in gas exchange. These findings are consistent with those of Hsia et al. [23] and Corrà et al. [24], but they differ from those of Beckers et al. [25], who compared 21 patients with HFrEF using both ergometers, finding a higher peak V^·^O_2_ on the treadmill, but without differences in ventilatory efficiency.

Due to the lack of inspiratory capacity maneuvers in the exercise modalities, the magnitude of dynamic hyperinflation could not be evaluated. In clinical practice, it is common to reserve the analysis of flow–volume curves and inspiratory capacity maneuvers for patients with unexplained dyspnea or patients with chronic respiratory diseases. The systematic quantification of dynamic hyperinflation is becoming increasingly important in the performance and interpretation of CPET. This phenomenon can occur in both vascular and respiratory pathologies, being of utmost importance in the latter group [26,27].

The differences in the hemodynamic response between exercise modalities in HF have been described by Kim et al. [28], who showed that treadmill exercise generates a higher cardiac output, reflected in increased V^·^O_2_ values. In our study, no significant differences were found between both groups (with and without COPD), probably due to a similar limitation in ejection fraction.

During follow-up, higher mortality was observed in the HFrEF-COPD group. COPD is independently associated with an increased risk of all-cause mortality and hospitalization in both heart failure with preserved ejection fraction and HFrEF, as demonstrated in previous studies [22,29].

Given the increase in the coexistence of COPD and HFrEF, it is crucial to manage patients with HFrFE-COPD via multidisciplinary healthcare teams, involving pulmonologists and cardiologists for optimal patient management.

The limitations of this study include the small number of patients, which is explained by the difficulty in selecting individuals with mixed pathology capable of performing a stress test. The differences in age and treatment between the two groups are another limitation to take into account. It should be noted that the initial test was performed for clinical purposes, which represents real life. Another limitation is the single-center nature of the study.

Among the strengths of our study is that this is the first study to investigate the response to effort in HFrEF-COPD patients based on the type of exercise. Despite this, we believe that the need to investigate cardiopulmonary interaction, their influence on exercise, and the potential clinical implications in sick populations justifies this study, with the aim of developing further research involving a greater number of patients and specialists.

## 5. Conclusions

In patients with HFrEF-COPD, treadmill CPET shows greater oxygen consumption and a lower ventilatory reserve, with a more pronounced ventilatory and cardiovascular response compared to the cycle ergometer. Cycle ergometer CPETs underestimate the ventilatory response to exercise, even in patients with confirmed respiratory pathology, which suggests the need to implement protocols that include the analysis of inspiratory capacity during the test.

When choosing the CPET and the exercise modality, it is key to assess the underlying pathologies and the clinical reason for the test.

Additionally, CPET should be adapted and its results interpreted by considering not only age, sex, and comorbidities but also the type of ergometer used.

## Figures and Tables

**Table 1:** General characteristics of both groups.

Variables	HFrEF (n = 47)	IHFrEF + COPD (n = 18)	*p*
**General Characteristics**			
**Age (years)**	**56.4 ± 9.6**	**61.6 ± 7.6**	**0.04**
Male sex	30 (70%)	16 (89%)	0.198
**BMI (kg/m^2^)**	**30 ± 4.2**	**27.1 ± 4.4**	**0.01**
LVEF (%)	29.9 ± 8.6	28.6 ± 8.6	0.599
**Lung function**			
**FEV1 (%)**	**88.5 ± 14.1**	**73.3 ± 14.8**	**0.001**
FVC (%)	90.6 ± 14.4	89.7 ± 19.3	0.864
**FEV 1/ FVC (%)**	**75.3 ± 3.8**	**62.4 ± 4.3**	**0.001**
**Functional class**			
NYHA class I	10 (21%)	3 (17%)	0.678
NYHA class II	29 (62%)	12 (66%)	0.711
NYHA class III	8 (17%)	3 (17%)	1000
**Cause of HFrEF**			
Ischemic	18 (39%)	10 (56%)	0.209
Idiopathic	20 (44%)	7 (39%)	0.788
Hereditary	6 (13%)	0 (0%)	0.175
Others	2 (4%)	1 (5%)	0.823

NS: not significant; BMI: body mass index; LVEF: left ventricular ejection fraction; FEV_1_(%): expiratory flow in the first second, percentage of predicted; FVC: forced vital capacity, percentage of predicted; NYHA: New York Heart Association.

**Table 2:** Chronic treatment for heart failure.

Chronic Treatment	HFrEF (n = 47)	HFrEF-COPD (n = 18)	*p*
**Beta-blocker**	45 (95.8%)	18 (100%)	0.520
**Amiodarone**	1 (2.1%)	2 (11.1%)	0.183
**RAASi**	32 (68%)	8 (44.4%)	0.411
**MRA**	25 (53.2%)	12 (66.7%)	0.407
**Valsartan/Sacubitril**	13 (27.7%)	9 (50%)	0.142
**Ivabradine**	5 (10.6%)	2 (11.1%)	0.956
**Loop diuretics**	30 (63.8%)	11 (61.1%)	0.839
**Thiazides**	47 (100%)	1 (5.6%)	0.277
**Implantable cardioverter** **defibrillator**			
Single chamber	7 (15%)	4 (22%)	0.693
CRT	12 (25.5%)	10 (56%)	0.03
No device	28 (59.6%)	4 (22%)	0.006

RAASi: renin-angiotensin-aldosterone system inhibitors, MRA: mineralocorticoid receptor antagonist, CRT: cardiac resynchronization therapy.

**Table 3:** Results of cardiopulmonary exercise test (CPET) parameters on the treadmill and cycle ergometer in patients with HFrEF-COPD.

	Treadmill Median (IQR)	Cycle Ergometer Median (IQR)	*p*
**Test duration (min^−1^)**	11 (7.5–12)	9 (8–10)	0.216
**V^·^** **O_2_ baseline ( mL/min^−1^)**	420 (330–550)	450 (370–545)	0.580
**V^·^** **O_2_ peak ( mL/min^−1^)**	1550 (1180–1820)	1225 (1040–1420)	<0.001
**V^·^O_2_ kg peak *(* mL/kg/ min^−1^)**	20 (17–23)	17 (15–19)	<0.001
**V^·^O_2_ at VT ( mL/min^−1^)**	1630 (1480–1710)	1290 (1070–1410)	<0.001
**V^·^CO_2_ at VT ( mL/min^−1^)**	1610 (1465–1685)	1275 (1040–1410)	<0.001
**RQ máx.**	0.9 (0.9–1)	1 (0.9–1)	0.880
**V^·^Emax (L/min^−1^)**	59 (48–62.5)	45.9 (38.6–46.8)	<0.001
**RR max (bpm^−1^)**	30.9 (26.5–36.4)	25.5 (21–28.5)	0.017
**BR%**	12 (2–35)	30 (22–48)	<0.001
**EQO_2_ at VT**	38 (34–39)	36.5 (33–39)	0.999
**EQCO_2_ at VT**	38 (35.5–39)	37 (33.5–39.5)	0.999
**VEslope**	32 (29–37)	35 (31–38.6)	0.042
**P_ET_CO_2_ baseline**	32 (30–36)	32.5 (31–34)	0.412
**P_ET_CO_2_ at VT (mmHg)**	35 (34–37)	37.5 (34–39)	0.249
**SatpO_2_ initial (%)**	96.5 (96–98)	98 (96–98)	0.007
**SatpO_2_ at the end (%)**	96 (95–98)	98 (97–98)	0.001
**HR baseline (lpm^−1^)**	71.5 (66.5–75)	68.5 (66.5–73.5)	0.348
**HR at the end (lpm^−1^)**	119 (102.5–132)	108.5 (100–120.5)	0.067
**HR recovery index (bpm^−1^)**	17 (12–25)	16 (14–19)	0.079
**V^·^O_2_ /FC (L/bpm/ min^−1^)**	14.1 (11.9–17.7)	12.7 (10.3–15.5)	<0.001
**SBP initial (mmHg)**	120 (110–140)	120 (115–140)	0.702
**SBP at the end (mmHg)**	170 (150–185)	162.5 (140–180)	0.562
**DBP initial (mmHg)**	70 (60–80)	80 (70–90)	0.075
**DBP at the end (mmHg)**	80 (70–90)	82.5 (80–90)	0.037
**Borg dyspnea at the end**	5.5 (5–7)	3.5 (2–4)	0.001
**Borg lower limbs at the end**	4 (0–7)	4 (3–5)	0.545

VT: ventilatory threshold; RQ: respiratory quotient; HR: heart rate; SBP: systolic blood pressure; DBP: diastolic blood pressure; V^·^O_2_: oxygen consumption; V^·^CO_2_: CO_2_ production; V^·^E: ventilation; RR: respiratory rate; EqO_2_: oxygen equivalent; EqCO_2_: carbon dioxide equivalent; P_ET_CO_2_: partial pressure end-tidal carbon dioxide; BR: breathing reserve; NS: not significant.

**Table 4:** Results of the cardiopulmonary exercise test in both groups (with and without COPD).

Parameters	HFrEF (n = 47) Median (IQR)	HFrEF+ COPD (n = 18) Median (IQR)	*p*
**Test Duration (minutes)**			
Treadmill	11 (9.1–12.5)	11 (7.5–12)	0.284
Cycle Ergometer	11 (9–12)	9 (8–10)	0.089
**Reaches VT (%)**			
Treadmill	95.7%	88.9%	0.305
Cycle Ergometer	78.7%	72.2%	0.578
**HR max. (bpm)**			
Treadmill	119 (108–131)	119 (102.5–132)	0.537
Cycle Ergometer	107 (99.5–120)	108.5 (100–120.5)	0.922
**HR recovery index (bpm)**			
Treadmill	20.5 (14–31)	17 (12–25)	0.463
Cycle Ergometer	15 (10–25)	16 (14–19)	0.902
**SBP at the End of Exercise (mmHg)**			
Treadmill	150 (137.5–170)	170 (150–185)	0.352
Cycle Ergometer	160 (140–180)	162.5 (140–180)	0.824
**DBP at the End of Exercise (mmHg)**			
Treadmill	80 (70–90)	80 (70–90)	0.549
Cycle Ergometer	90 (80–100)	82.5 (80–90)	0.417
**Final O_2_ Saturation (%)**			
Treadmill	97 (96–98)	98 (97–98)	0.380
Cycle Ergometer	97 (96–98)	96 (95–98)	0.294
**V^·^O_2_ peak (ml/min)**			
Treadmill	1690 (1425–2140)	1550 (1180–1820)	0.101
Cycle Ergometer	1390 (1190–1680)	1225 (1040–1420)	0.125
**V^·^O_2_ /HR max. (ml/bpm/ min^−1^)**			
Treadmill	14.1 (11.8–17.1)	14.1 (11.9–17.7)	0.675
Cycle Ergometer	12.1 (9.7–15.2)	12.7 (10.3–15.5)	0.806
**V^·^E Slope**			
Treadmill	29 (27–34)	32 (29–37)	0.066
Cycle Ergometer	**32 (27–35.8)**	**35 (31–38.6)**	**0.042**
**V^·^E max. (L/ min^−1^)**			
Treadmill	53.3 (46.5–65.5)	59 (48–62.5)	0.765
Cycle Ergometer	45.8 (37.9–51.8)	45.9 (38.6–46.8)	0.686
**RR max (rpm^−1^)**			
Treadmill	30 (25.3–34.9)	30.9 (26.5–36.4)	0.804
Cycle Ergometer	27 (24.9–32.5)	25.5 (21–28.5)	0.076
**EqO_2_ at VT**			
Treadmill	**35 (32–38)**	**38 (34–39)**	**0.049**
Cycle Ergometer	36.5 (33–41)	36.5 (33–39)	0.919
**EQCO_2_ at VT**			
Treadmill	**35.5 (32.5–38.5)**	**38.8 (35.5–39)**	**0.042**
Cycle Ergometer	37 (33–41.5)	37 (33.5–39.5)	0.671
**P_ET_ CO_2_ at VT (mmHg)**			
Treadmill	36.5 (33.5–39.5)	35 (34–37)	0.075
Cycle Ergometer	35.5 (32–39)	37.5 (34–39)	0.958
**ΔP_ET_CO_2_ (mmHg)**			
Treadmill	4.5 (1–6)	3.5 (3–5.5)	0.518
Cycle Ergometer	2 (0–5)	3 (1.5–3.5)	0.730
**BR at max. exercise (%)**			
Treadmill	**30 (25.3–34.9)**	**12 (2–35)**	**0.006**
Cycle Ergometer	**27 (24.9–32.5)**	**30 (22–48)**	**0.020**
**EOV (%)**			
Treadmill	**41.3%**	**0%**	**0.001**
Cycle Ergometer	**45.6%**	**11.8%**	**0.010**
**Final Borg Dyspnea (1–10)**			
Treadmill	6 (3.5–7)	5.5 (5–7)	0.567
Cycle Ergometer	4 (2–5.5)	3.5 (2–4)	0.561
**Final Borg Lower Limbs (1–10)**			
Treadmill	3 (0.5–7)	4 (0–7)	0.861
Cycle Ergometer	5 (4–7)	4 (3–5)	0.178

NS: not significant; COPD: chronic obstructive pulmonary disease; VT: Ventilatory Threshold; RQ: respiratory quotient; HR: heart rate; SBP: systolic blood pressure; DBP: diastolic blood pressure; V^·^O_2_: oxygen consumption; V^·^CO_2_: CO_2_ production; EqO_2_: oxygen equivalent; EqCO_2_: carbon dioxide equivalent; P_ET_ CO_2_: partial pressure end-tidal carbon dioxide; ΔP_ET_CO_2_: P_ET_CO_2_ increase from start of test to VT; BR: breathing reserve; EOV: exercise oscillatory ventilation.

## Data Availability

The original contributions presented in this study are included in the article. Further inquiries can be directed to the corresponding author(s).
